# Anatomy and Physiology

**Published:** 1837-07

**Authors:** 


### PART FOURTH.

### Selections from the British Journals.

(FOR THE QUARTER ENDING APRIL 30, 1837.)

---

### ANATOMY AND PHYSIOLOGY.

### *A Description of the Pulmonic Pulse.* By P. MOLLISON, M.D.

THIS term Dr. Mollison applies to a phenomenon which "consists in the expulsion of a certain quantity of air in the chest, synchronously with each contraction of the heart." The fact of such expulsion he ascertained by inserting into one nostril a bent glass tube, open at both ends, with one leg shorter than the other, and suspending the respiration. "Upon examining the water in the tube, it was seen moving backwards and forwards, a distance of five-sixteenths of an inch, in a manner analogous to the pulse at the wrist, and synchronous with it." Generally the fluid is driven *from* the lungs; in some cases it is driven *towards* them. The cause of this phenomenon Dr. M. considers to be "the diastole of the branches of the pulmonary arteries, which, running side by side with the bronchial ramifications, will readily compress them to a certain extent, and thus expel a portion of their contained air." It remains to be seen whether this observation is capable of any useful application either in physiology or practice.

*British Annals of Medicine, March* 17, 1837.

---

### *Physiological Observations on the Pulsations of the Heart, and on its Diurnal Revolution and Excitability.* By Dr. KNOX.

THIS is a very interesting and important paper, containing numerous original observations, and leading to conclusions, not merely interesting in a physiological point of view, but which may be found useful in the practice of medicine. In the year 1815, Dr. Knox published, in the Edinburgh Journal, an essay on the same subjects, of which the present paper may be regarded as a continuation. We regret that our limits prevent us from giving in this place more than a few of the results of the individual experiments and the general conclusions:

Dr. Knox contends that there can be no such thing as an *average pulse* in man, seeing that this varies every hour of the day and night, and after every meal, and is extensively influenced by merely rising from the sitting to the erect posture. Some data, however, existed on this point before Dr. Knox, and some are added by him. Bryan Robinson gives the average pulse of two men, at every hour of the day, (whilst sitting,) from eight A.M. to eleven P.M., taken for several weeks; the mean of the one 76, and of the other 78. Dr. Knox gives the mean pulse of twenty-five young gentlemen, taken between the hours of twelve and two: it was 72.4 sitting, and 75.4 standing; their mean age was twenty-five. The pulse in infants and young children was found by Dr. Knox too variable to lead to any decided result. The following are some of the results observed by M. Billard: in forty-one infants, from one to ten days' old, the pulse varied from 80 to 180; in eighteen of these it was less than 80, and in ten between 110 and 130. In thirty-six children, from one to two months, it varied from 80 to 150; in fourteen of these it was from 80 to 85, and in seven from 125 to 130. In twenty children, from two to three months: in fourteen it was more than 90, and in four from 70

to 80. M. Quetelet found the average pulse of thirty-six infants, at birth, one-half of each sex, to be as follows:—*Max.* 165, *Min.* 106, *Med.* 135.

It is extremely difficult to reckon accurately the normal respiration, and, consequently, the relation of the inspirations to the pulse. The most accurate observations are those of Quetelet, who gives the following results obtained from 300 males of different ages:

| | Birth to 5 yrs. | From 5 to 10 | From 10 to15 | From 15 to20 | From 20 to25 | From 25 to30 | From 30 to50 |
|---|---|---|---|---|---|---|---|
| Mean number of pulsations ...... | 136 | 88 | 78 | 69 | 69 | 71 | 70 |
| inspirations ...... | 44 | 26 | | 20 | 18 | 16 | 18 |

The pulsation and respiration vary, both absolutely and relatively, during waking and sleep. The following are some of the average results obtained by Quetelet on children and women:—*Pulsations,* awake, 91; asleep, 78: *Inspirations,* awake, 28; asleep, 22.

The difference between the frequency of the pulse in the different postures of the body has been long known, as also the variation of the pulse with the time of the day, exercise, &c. We apprehend, however, that the general opinions are very incorrect on these points, and will be set right by several of Dr. Knox's observations. In the case of a gentleman, aged twenty, of the most regular habits, the following are the results of a week's observation of the pulse:—*Average morning pulse,* lying, 62; sitting, 78; standing, 90. *Average evening pulse,* lying, 56; sitting, 67; standing, 77; making what has been termed the *differential pulse* (or the difference between the pulse in the lying and standing posture,) twenty-eight in the morning and twenty-one in the evening. The same gentleman was made the subject of another series of observations for the space of fourteen days, and the following are the average results obtained: Pulse, at seven A.M. (in bed,) 58.5; at nine A.M. (sitting and writing,) 94.6; at eleven A.M. (sitting at lecture,) 82.6; at one P.M. (standing,) 83; at three P.M. (sitting at lecture,) 74.9; at five P.M. (sitting at home,) 72.9; at seven P.M. (ditto,) 73.6; at nine P.M. (ditto,) 71.6; at eleven P.M. (ditto,) 65.7. During these observations, breakfast was taken at half-past seven, dinner at half-past five, coffee at half-past eight; and no wine, spirits, or malt liquor was drunk.

Several tables are given to illustrate the effects of exercise. The following numbers represent the average of three observations made on a young gentleman, *after* walking fast, viz.: 1st, one mile in a quarter of an hour; 2d, four miles in one hour; 3d, one mile in ten minutes. *Pulse after the walk:* after first five minutes, 106; second ditto, 96; third ditto, 86; fourth ditto, 89; fifth ditto, 87; sixth ditto, 87; seventh ditto, 84.

The following are Dr. Knox's *general conclusions,* which, however, he very modestly wishes to be considered as only *his own;* leaving it to his readers to deduce from his facts such others as they may seem to them to warrant.

"1. The velocity of the heart's action is in the direct ratio of the age of the individual, being quickest in young persons, slowest in the aged. There may be exceptions to this, but they do not affect the general law.

"2. There are no data to determine the question of an average pulse for all ages.

"3. There is a morning acceleration and an evening retardation in the number of the pulsation of the heart, independent of any stimulation by food, &c.

"4. The excitability of the heart undergoes a daily revolution; that is, food and exercise most affect the heart's action in the morning and during the forenoon, least in the afternoon, and least of all in the evening. Hence we should infer that the pernicious use of spirituous liquors must be greatly aggravated in those who drink before dinner.

"5. Sleep does not farther affect the heart's action than by a cessation of all voluntary motion, and by a recumbent position.

"6. In weak persons, muscular action excites the action of the heart more powerfully than in strong and healthy individuals; but this does not apply to other stimulants, to wine, for example, or to spirituous liquors.

"7. The effects of the position of the body in increasing or diminishing the number of pulsations is solely attributable to the muscular exertion required to maintain the body in the sitting or erect position; the debility may be measured by altering the position of the person from a recumbent to the sitting or to the erect position.

"9. The law of the differential pulse is not universal. There are exceptions to be found even in those in perfect health. It is also possible that there may be some in whom the diurnal revolutions of the pulse takes place only in consequence of the use of stimulants. But this has not been proved satisfactorily.

"10. The most powerful stimulant to the heart's action is muscular exertion. The febrile pulse never equals this.

"11. The law of relation between the inspiration and pulsation of the heart has been stated by M. Quetelet." *Edinburgh Journal. April*, 1837.

